# Quantitative ^166^Ho-microspheres SPECT derived from a dual-isotope acquisition with ^99m^Tc-colloid is clinically feasible

**DOI:** 10.1186/s40658-020-00317-8

**Published:** 2020-07-14

**Authors:** M. Stella, AJAT Braat, MGEH Lam, HWAM de Jong, R. van Rooij

**Affiliations:** Department of Radiology and Nuclear Medicine, University Medical Center, Utrecht, Heidelberglaan 100, 3584 CX Utrecht, The Netherlands

**Keywords:** Radioembolization, Holmium-166, SPECT/CT, Dual isotope, Technetium, Scatter correction

## Abstract

**Purpose:**

Accurate dosimetry is essential in radioembolization. To this purpose, an automatic protocol for healthy liver dosimetry based on dual isotope (DI) SPECT imaging, combining holmium-166 (^166^Ho)-microspheres, and technetium-99 m (^99m^Tc)-colloid was developed: ^166^Ho-microspheres used as scout and therapeutic particles, and ^99m^Tc-colloid to identify the healthy liver. DI SPECT allows for an automatic and accurate estimation of absorbed doses, introducing true personalized dosimetry. However, photon crosstalk between isotopes can compromise image quality. This study investigates the effect of ^99m^Tc downscatter on ^166^Ho dosimetry, by comparing ^166^Ho-SPECT reconstructions of patient scans acquired before (^166^Ho-only) and after additional administration of ^99m^Tc-colloid (^166^Ho-DI).

**Methods:**

The ^166^Ho-only and ^166^Ho-DI scans were performed in short succession by injecting ^99m^Tc-colloid on the scanner table. To compensate for ^99m^Tc downscatter, its influence was accounted for in the DI image reconstruction using energy window-based scatter correction methods. The qualitative assessment was performed by independent blinded comparison by two nuclear medicine physicians assessing 65 pairs of SPECT/CT. Inter-observer agreement was tested by Cohen’s kappa coefficient. For the quantitative analysis, two volumes of interest within the liver, VOI_TUMOR_, and VOI_HEALTHY_ were manually delineated on the ^166^Ho-only reconstruction and transferred to the co-registered ^166^Ho-DI reconstruction. Absorbed dose within the resulting VOIs, and in the lungs (VOI_LUNGS_), was calculated based on the administered therapeutic activity.

**Results:**

The qualitative assessment showed no distinct clinical preference for either ^166^Ho-only or ^166^Ho-DI SPECT (kappa = 0.093). Quantitative analysis indicated that the mean absorbed dose difference between ^166^Ho-DI and ^166^Ho-only was − 2.00 ± 2.84 Gy (median 27 Gy; *p* value < 0.00001), − 5.27 ± 8.99 Gy (median 116 Gy; *p* value = 0.00035), and 0.80 ± 1.08 Gy (median 3 Gy; *p* value < 0.00001) for VOI_HEALTHY,_ VOI_TUMOR,_ and VOI_LUNGS_, respectively. The corresponding Pearson’s correlation coefficient between ^166^Ho-only and ^166^Ho-DI for absorbed dose was 0.97, 0.99, and 0.82, respectively.

**Conclusion:**

The DI protocol enables automatic dosimetry with undiminished image quality and accuracy.

**Clinical trials:**

The clinical study mentioned is registered with Clinicaltrials.gov (NCT02067988) on 20 February 2014**.**

## Introduction

Over the past decade, the number of radioembolization procedures in the treatment of liver-only or liver-dominant hepatic malignancy has rapidly increased [1]. Radioembolization is a catheter-based therapy that delivers internal radiation to tumors. Currently, three devices are commercially available: SIR-Spheres® (SIRTeX Medical Ltd.), TheraSphere® (BTG Ltd./Boston Scientific), both loaded with yttrium-90 (^90^Y), and QuiremSpheres® (Quirem Medical B.V.), loaded with holmium-166 (^166^Ho). Radioembolization requires a comprehensive initial safety evaluation (identifying potential non-target tissue irradiation) and assessment of intrahepatic microspheres distribution for dosimetric evaluation. Pre-treatment image-based dosimetry enables radioembolization optimization, because it allows assessment of the biodistribution of microspheres in the liver, which is often heterogeneous and clustered. Because absorbed dose and treatment outcome (toxicity and efficacy) are correlated, dosimetry should ultimately lead to improved patient selection and individualized treatment planning [2]. For this reason, prior to treatment, either ^99m^Tc-MAA or a ^166^Ho-scout dose (QuiremScout®, Quirem Medical B.V.) is administered to simulate the actual treatment. ^166^Ho-microspheres may be preferred as simulation particles (i.e., scout dose), because they are identical to the treatment particles, which makes them superior in the prediction of the treatment dose distribution [3, 4]. Additionally, ^166^Ho allows for quantitative SPECT analysis and consequently dosimetric assessment [5].

For personalized treatment planning, several dosimetric thresholds need to be determined: (1) the minimum required tumor radiation absorbed dose to obtain an adequate tumor response, (2) an acceptable healthy liver tissue absorbed dose to limit post-treatment toxicities, and (3) the maximum tolerable lung shunt dose to prevent radiation pneumonitis. Obtaining these dosimetric values requires delineation of the liver, tumors, and lungs using anatomical images such MRI and CT. Accurate tumor and healthy liver delineation (segmentation) and image co-registration are challenging. Segmentation is usually done manually, which is time-consuming and user-dependent. Registration between anatomical images (MRI or contrast-enhanced CT), acquired hours to weeks prior to the treatment, and the functional image (SPECT) following the scout procedure is challenging due to interval deformations of the liver. Therefore, a dual-isotope SPECT/CT protocol was developed to improve dosimetry [6], having the potential to allow for the automatic delineation of tumor and healthy liver, and obviating the need for co-registration. To this end, a ^166^Ho-scout dose for treatment simulation is followed by intravenously injected colloid (^99m^Tc-stanneous phytate, PHYTACIS® by Curium Pharma, Petten, The Netherlands). The colloid accumulates in Kupffer cells, presents in healthy liver tissue, and absent in tumorous tissue [7]. It allows for automatic normal liver tissue segmentation by thresholding the ^99m^Tc image. This dual-isotope protocol enables the automatic estimation of the healthy tissue absorbed dose, which is considered to be the major dose-limiting factor. It facilitates performing dosimetry in every patient, which may lead to an improved and more personalized prescribed activity, avoiding over-dosing or, even more frequently, under-dosing the target, sacrificing efficacy for safety [8]. Dual isotope (DI) SPECT however comes with the technical challenge of correcting for the crosstalk between the two isotopes: scatter from ^99m^Tc contaminating the main ^166^Ho energy window and vice versa.

In previous work, van Rooij et al. [9] demonstrated the technical feasibility of quantitative ^166^Ho SPECT reconstructions in the presence of ^99m^Tc in a phantom study. These reconstructions were obtained using the in-house developed Monte Carlo SPECT reconstruction software (UMCS). However, a systematic comparison between ^166^Ho-DI SPECT and ^166^Ho-only SPECT using patient data reconstructed using a commercially available software is required to consider this DI concept for clinical practice. For this reason, a qualitative and quantitative comparison between ^166^Ho-only acquisitions and ^166^Ho acquisitions in presence of ^99m^Tc was investigated.

## Materials and methods

### Study population

For all SPECT/CT acquisitions used in this study, informed consent was obtained as part of the HEPAR PLuS study [10]. Thirty-one patients with liver metastases of neuroendocrine tumors were analyzed, 29 scout (pre-treatment) procedures (average administered activity 208 ± 52 MBq) and 36 therapeutic treatments (average administered activity 5757 ± 2716 MBq). Baseline characteristics of these patients are presented in Table 1. Two subjects were excluded because of the impossibility of segmenting the minimum desired volumes of interest (25 ml) in compliance with the defined resolution requirements for a proper absorbed dose estimate using SPECT. This constrain was introduced in order to limit the errors related to registration and dosimetry quantification accuracy, affected by small volume definition. According to the mentioned study protocol, for each of the 65 procedures considered, two SPECT/CT images were acquired after the activity injection, a ^166^Ho-only and ^166^Ho-DI SPECT. According to the image acquisition protocol, all scans were performed when the total activity at the scanning time was approximately 250 MBq, enabling a comparison between pre and post-treatment images.

**Table 1:** Characteristics of patients with neuroendocrine liver metastases treated in the HEPAR PLuS trial, included in this study

Characteristics	N or median	
N patient	29	
Pre-treatment Post-treatment	26 32^‡^	
Sex		
Male Female	21 8	
Age (years)^*^	63 ± 8	
Treatment type	Pre-treatment	Post-treatment
Whole liver Partial liver	21^†^ 5	15^†^ 17

^*^At first treatment

^†^After right side hemi hepatectomy

‡Multiple radioembolization treatment based on the same scout procedure

### Image acquisition

**Fig. 1:**
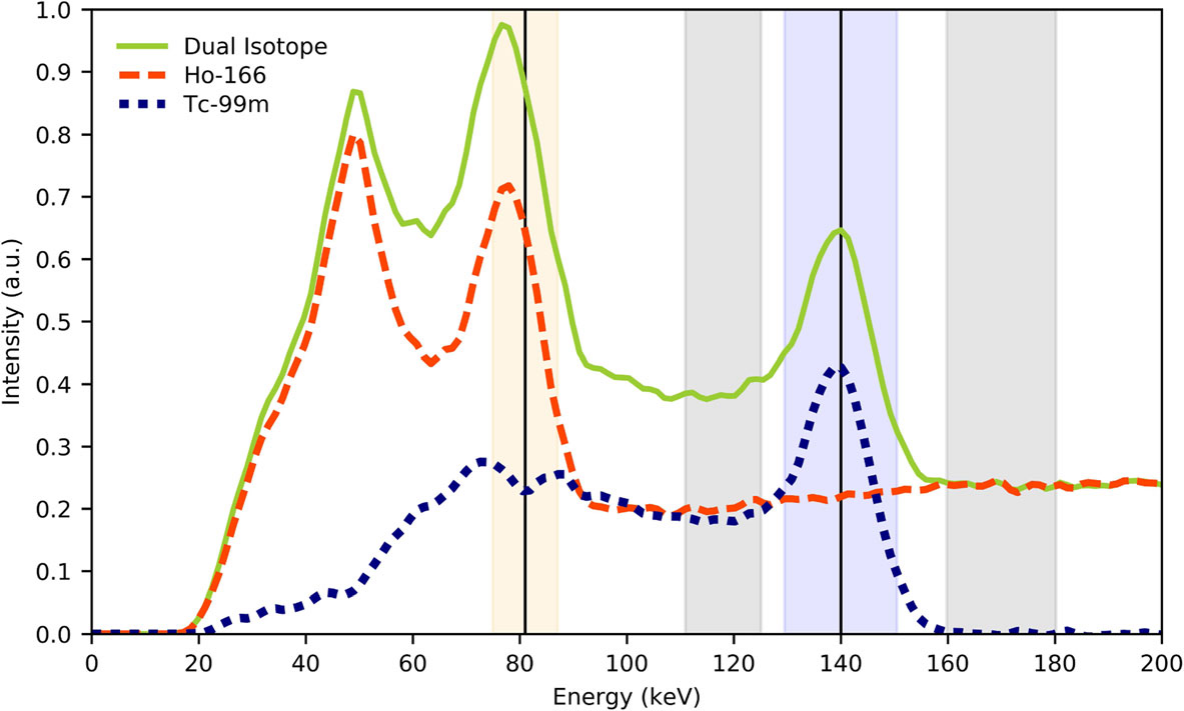
Dual isotope energy spectrum. The solid black vertical lines at 80.6 keV (within the orange window) and 140 keV (within the blue window) denote the ^166^Ho and ^99m^Tc photopeak, respectively. The solid green curve is the dual isotope spectrum, recorded when both ^166^Ho and ^99m^Tc were present, and the dashed orange curve represents the ^166^Ho spectrum. The dotted blue curve was obtained by subtracting the ^166^Ho spectrum from the dual isotope spectrum and represents the ^99m^Tc spectrum. Energy windows used to estimate the downscatter correction (centered at 118 keV, 12% width and at 170 keV, 12% width) are indicated in gray

All patients were scanned on a Symbia T16 dual head SPECT/CT scanner (Siemens, Erlangen, Germany), using a medium-energy low-penetration collimator, on a 128 × 128 matrix (pixel spacing, 4.8 × 4.8 mm), with 120 angles (15 s per projection) over a non-circular 360° orbit. An energy window centered at the 81 keV photopeak with a width of 15% was used for both ^166^Ho-only and ^166^Ho-DI acquisitions (see Fig. 1). An additional energy window centered at 118 keV (12% width) was used to correct the ^166^Ho photopeak data for downscatter using a window-based scatter correction [11]. ^99m^Tc was imaged using a 140 keV, 15% wide, energy window, with an upper scatter window at 170 keV (12% width) to correct for ^166^Ho downscatter. The first SPECT/CT was acquired after the intra-arterial injection of ^166^Ho-microspheres (^166^Ho-only SPECT), while the second SPECT/CT was acquired 10 min after additional 50 MBq ^99m^Tc-stannous phytate injection (^166^Ho-DI SPECT). To minimize patient motion, ^99m^Tc-stannous phytate was administered while the patient remained on the SPECT/CT table in supine position. The optimal ^166^Ho-^99m^Tc ratio, yielding a high accuracy in DI reconstruction, was previously empirically determined by van Rooij et al. [9] in our institution, based on phantom data, and the resulting 5:1 ^166^Ho-^99m^Tc activity ratio was adopted for this study.

### SPECT reconstruction

SPECT images were reconstructed using a 3D OSEM algorithm (Flash 3D; Siemens) with 10 iterations, 8 subsets, incorporating attenuation correction. To correct for scatter during the reconstruction of the ^166^Ho activity distribution, downscatter in the 81 keV photopeak window due to higher energy emissions of both ^166^Ho and ^99m^Tc was estimated from the 118 keV energy window by applying a single combined k-factor of 1.15 (see Additional file 1: Supplemental material for details). Photopeak scatter, i.e., scattered photons originating from the 81 keV primary photopeak, was not accounted for.

### Qualitative analysis

**Fig. 2:**
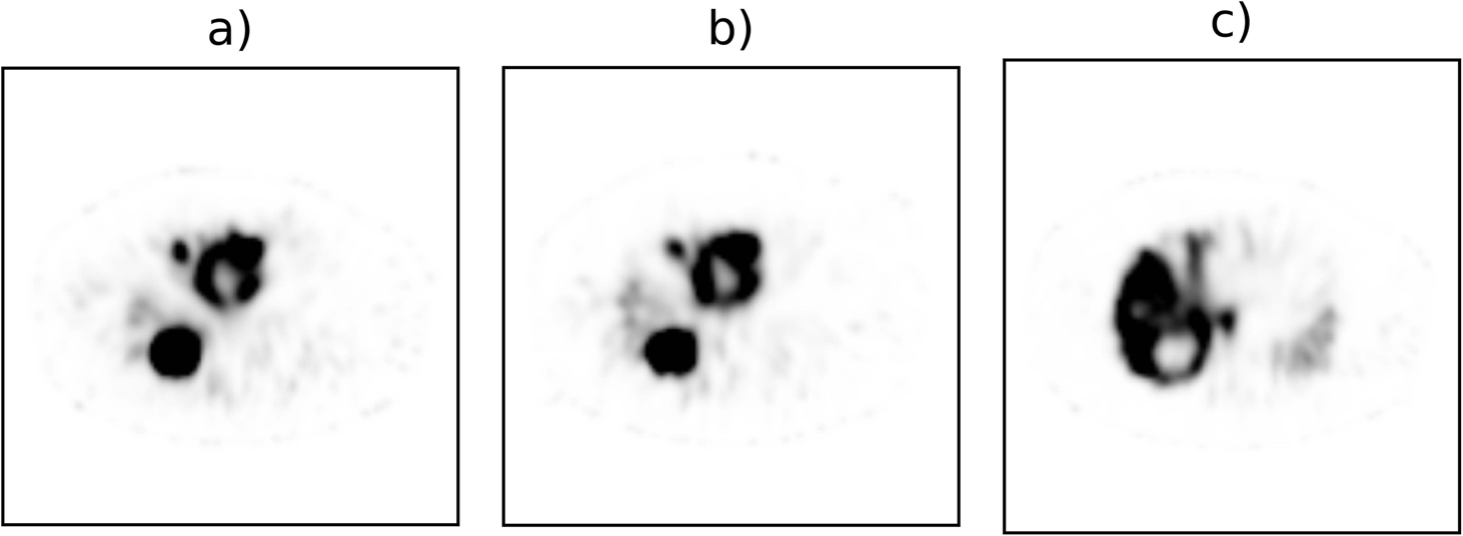
SPECT images of a 69-year-old male with neuroendocrine tumor in the pancreas. The patient was diagnosed with metastatic spread to the liver, which was treated with a ^166^Ho radioembolization procedure in the whole liver (prescribed activity 9900 MBq). **a**^166^Ho-DI and **b**^166^Ho-only acquisition images have been, independently and blindly, presented to the nuclear medicine physicians for the qualitative assessment. **c**^99m^Tc image acquired during the DI protocol where an additional 50 MBq of ^99m^Tc-colloid was administered

For the qualitative assessment, 65 pairs of SPECT/CT reconstructions (^166^Ho-only and DI) were considered, divided into 29 scout dose SPECTs and 36 post-treatment SPECTs, and a Gaussian filter with σ = 4.2 mm was applied to reduce the noise. Two nuclear medicine physicians (M.L. and A.B., > 5 years’ experience) were randomly and blindly presented each pair of acquisitions (an example is depicted in Fig. 2). Then, they were independently asked to express clinical preference for either ^166^Ho-only or DI, and whether both acquisitions could be considered clinically acceptable for diagnostic purpose or not.

### Quantitative analysis

To allow for a comparison between pre- and post-treatment data, all SPECT images were scaled to units of Bq/ml, in such a way that for each image, the total activity matched the administered treatment activity, based on the assumption that the entire activity was present in the reconstructed field of view. Under the general assumption that microspheres remain lodged long enough for their entire activity to decay, the absorbed radiation dose in a VOI can be calculated as:

$$ Dose\left[ Gy\right]=15.87\left[\frac{mJ}{MBq}\right]\frac{\  Activity\ concentration\ \left[ Bq/\mathrm{ml}\right]\ast {10}^{-6}}{VOI\  density\left[g/\mathrm{ml}\right]} $$

where 15.87 mJ/MBq represents the deposited energy due to the β decay of 1 MBq initial ^166^Ho activity. For the liver, a soft tissue density of 1.06 g/cm^3^ was applied [12], while the lung density value was set to 0.3 g/cm^3^ [13], assuming, for both organs, a homogenous organ density value, constant among patients. Since the mean penetration of the β emission of ^166^Ho (2.5 mm) is small compared to the voxel size (4.8 mm), all energy was assumed to be absorbed within the considered voxel [4]. The β radiation accounts for 96% of the emitted energy (=15.87 mJ/MBq), and the other 4% of the energy is for the most part emitted through γ radiation. Because of the relatively large penetration distance of these γ and the inverse square law, the absorbed radiation dose due to γ emissions was ignored in this study.

**Fig. 3:**
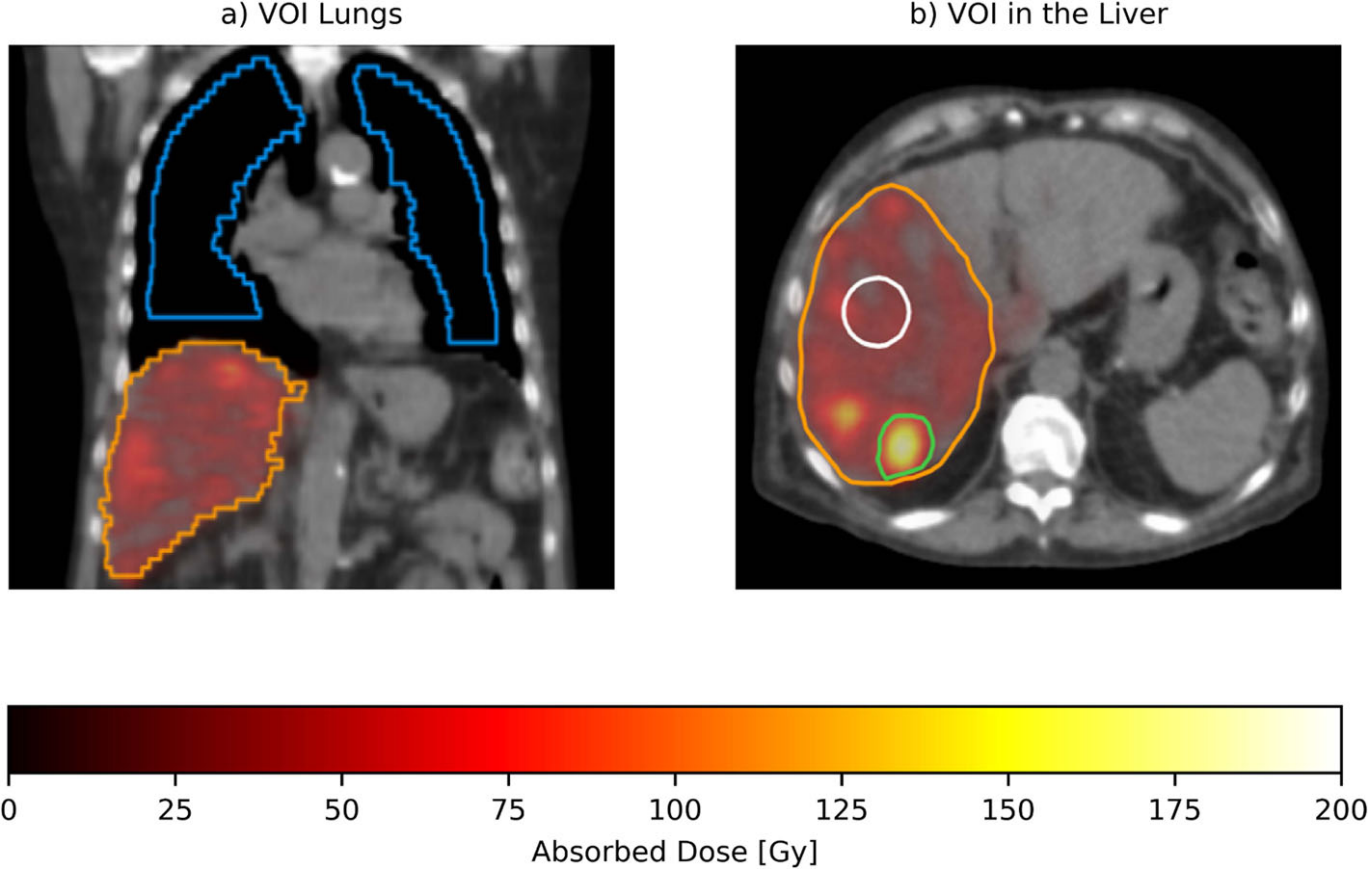
^166^Ho-SPECT/CT images of a 70-year-old male diagnosed with multiple liver metastases of neuroendocrine origin after receiving a ^166^Ho radioembolization treatment (right liver lobe, 4207 MBq). **a** Coronal view where the orange line defines the treated liver, and the blue line depicts the semi-automatically delineated lungs, after the shrinkage process. **b** Axial view of the liver with 3 VOIs superimposed. Orange line delineates the treated liver (right lobe), and green line delineates the tumor (VOI_TUMOR_) while white line defines the VOI_HEALTHY_

For the assessment of the mean absorbed dose in the liver, all of the ^166^Ho-DI SPECT/CT images were co-registered with the corresponding ^166^Ho-only SPECT/CT images to compensate for possible patient movement during the time lag between the two acquisitions (10 min to account for ^99m^Tc-stannous phytate injection and distribution). Since the patient remained on the table, a rigid registration was performed. The registration was carried out with Elastix [14], based on the SPECT related LDCT (primarily used to compute the attenuation correction map), using an adaptive stochastic gradient descendent approach as optimizer and a mutual information metric. Subsequently, two volumes of interest (VOIs) for each pair of acquisitions were manually defined on the ^166^Ho-only SPECT/CT: VOI_HEALTHY_ (3D ellipsoidal shape within the healthy liver) and VOI_TUMOR_ (one manually segmented tumor among the multiple tumors present), as depicted in Fig. 3b. For both VOI_HEALTHY_ and VOI_TUMOR_, it has been decided to constrain the minimum volume to 25 ml, to ensure a reliable activity recovery. These VOIs were applied to the co-registered ^166^Ho-DI SPECT to compute the mean absorbed dose within these VOIs for comparison.

To estimate the mean lung shunt dose, the lungs were semi-automatically delineated on both the corresponding ^166^Ho-only CT and ^166^Ho-DI CT images with the Q-suite™ software (Quirem Medical B.V.). Before the lung masks were applied to the SPECT reconstructions for the absorbed dose computation, the delineations were shrunk by 2 cm to avoid any partial volume effect close to the edges and to minimize the influence of scatter from activity in the liver.

The mean absorbed dose, expressed in gray [Gy], was chosen as the metric for comparison since it was deemed the most clinically relevant parameter. The dose difference between ^166^Ho-DI and ^166^Ho-only was reported, since it was considered more relevant from a clinical point of view than a relative measurement (e.g., percentage difference).

### Statistical analyses

In each comparative analysis, the ^166^Ho-only SPECT/CT images were considered as reference standard. Inter-observers’ agreement was measured by means of Cohen’s kappa statistic (*κ*) [15], for the qualitative assessment. For this analysis, it was assumed that ^166^Ho-only and ^166^Ho-DI SPECT/CT data were paired, as both scans were acquired within a short time interval (< 10 min), with patient and bed table in the same position. Pre and post-treatment results were also independently reported for completeness of the data presented. To compare the absorbed dose in the VOIs between ^166^Ho-only and ^166^Ho-DI, after a visual assessment for data normality, Bland-Altman analyses were performed. For each plot, the mean absorbed dose difference between ^166^Ho-DI and ^166^Ho-only, expressed in Gy, and limits of agreements (LoA), [Gy], were reported. LoA are computed as mean ± coefficient of reproducibility (CRP), equal to 1.96 × standard deviation. The linear correlation between absorbed dose in ^166^Ho-only and ^166^Ho-DI was expressed in terms of the Pearson’s correlation coefficient *r*. In addition, two-sided paired *t* test (at *α* = 0.05) was performed to check the statistical difference between mean absorbed dose based on ^166^Ho-only and on ^166^Ho-DI SPECT (null hypothesis is no difference between mean absorbed dose values computed on ^166^Ho-only and on ^166^Ho-DI SPECT).

## Results

### Qualitative analysis

According to the qualitative assessment carried out by two expert nuclear medicine physicians, all ^166^Ho-SPECT reconstructions were considered reliable for a diagnostic purpose. Based on their preference for either ^166^Ho-only or ^166^Ho-DI, their inter observer agreement (Cohen’s kappa coefficient) was equal to 0.09 (*κ*= − 0.63 and *κ*= 0.14 for pre-treatment and post-treatment data, respectively). The Cohen’s *κ* value, close to 0, shows the lack of agreement on a favorite imaging option (^166^Ho-only or ^166^Ho-DI). Moreover, both were considered suitable for diagnostic use. Therefore, no distinct preference for either one of the reconstructions for use in clinical practice could be concluded.

### Quantitative analysis

The constraint in volume for the VOIs (≥ 25 ml) led to the exclusion of 21 procedures for the VOI_TUMOR_ and of 7 procedures for the VOI_HEALTHY_, since they did not satisfy this requirement. The lower limit for the VOI volume was introduced to ensure an adequate dose recovery. The included dataset is reported in Table 2.

**Table 2:** Quantitative dataset characteristics. (a) Top row reports the number of VOI_HEALTHY_ and related volume value (mean and standard deviation) for both pre- and post-treatment dataset, and the middle row refers to VOI_TUMOR_; while the bottom row illustrates the data related to VOI_LUNGS_ with volumes values after the shrinkage process for both ^166^Ho-only and ^166^Ho-DI. b) median and quartile deviation of mean absorbed dose recovered in VOIs, for both ^166^Ho-only and ^166^Ho-DI are reported. First row refers to VOI_HEALTHY_, second to VOI_TUMOR_, and third to VOI_LUNGS_, respectively

**(a)**	**Pre-treatment**		**Post-treatment**	
VOI	Number	Volume	Number	Volume
Healthy	26	27.57 ml	32	27.57 ml
Tumor	21	27.50 ml ± 3.91	23	26.27 ml ± 2.23
Lungs	26	2399.30 ml ± 971.77 (^166^Ho-Only) 2182.47 ml ± 794.58 (^166^Ho-DI)	31	2578.09 ml ± 1026.67 (^166^Ho-Only) 2263.47 ml ± 841.22 (^166^Ho-DI)
**(b)**	^**166**^**Ho-only**		^**166**^**Ho-DI**	
VOI	Median	Quartile deviation (IQR/2)	Median	Quartile deviation (IQR/2)
Healthy	27.12 Gy	7.08 Gy	26.22 Gy	8.00 Gy
Tumor	116.27 Gy	44.91 Gy	108.96 Gy	46.00 Gy
Lungs	2.99 Gy	0.99 Gy	3.88 Gy	1.07 Gy

The results, for each of the three VOIs, for both the dataset considered entirely and split among pre- and post-treatment data, are reported in Table 3.

**Table 3:** Mean absorbed dose difference (^166^Ho-DI and ^166^Ho-only), standard deviation, and lower and upper limits of agreement for VOIs of interest (VOI_HEALTHY_, VOI_TUMOR_, and VOI_LUNGS_). Top row depicts values for the dataset considered entirely, while middle and bottom rows report values for pre- and post-treatment data, respectively

		VOI_**HEALTHY**_	VOI_**TUMOR**_	VOI_**LUNGS**_
**All data**	Average ± SD [Gy]	− 2.00 ± 2.84	− 5.27 ± 8.99	0.80 ± 1.08
	Limits of agreement [Gy]	− 7.56 + 3.56	− 22.89 +12.35	− 1.32 + 2.92
**Pre-treatment data**	Average ± SD [Gy]	− 2.69 ± 3.23	− 4.51 ± 8.69	0.86 ± 1.24
	Limits of agreement [Gy]	− 9.02 + 3.64	− 21.54 + 12.52	− 1.57+ 3.29
**Post-treatment data**	Average ± SD [Gy]	− 1.44 ± 2.27	− 5.96 ± 9.01	0.74 ± 0.92
	Limits of agreement [Gy]	− 5.89 + 3.01	− 23.62 + 11.70	− 1.06 + 2.54

**Fig. 4:**
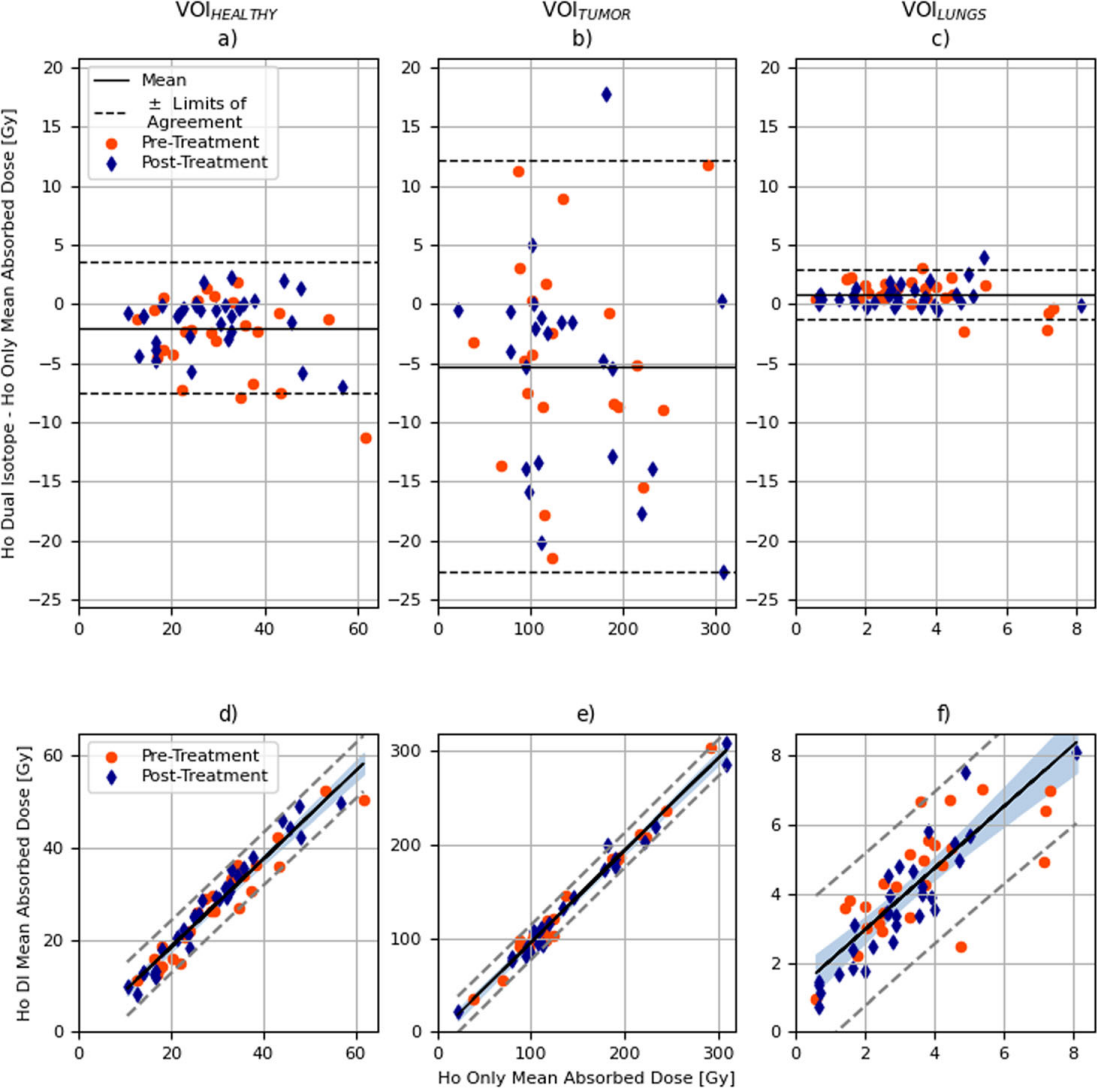
Bland-Altman plot (difference between mean absorbed dose recovered in ^166^Ho-DI and in ^166^Ho-only against mean absorbed dose in ^166^Ho-only) is depicted in the top row. Orange circles refer to pre-treatment dataset while blue diamonds to post-treatment. Mean of difference between absorbed dose recovered in ^166^Ho-DI and in ^166^Ho-only (${\overline{dose}}_{166\mathrm{Ho}-\mathrm{DI}}-{\overline{dose}}_{166\mathrm{Ho}-\mathrm{only}}$) is depicted by the black solid line, while black dashed lines define ± limits of agreement. Data in (**a**) refers to VOI_HEALTHY_, in (**b**) to VOI_TUMOR_, and (**c)** depicts data referring to VOI_LUNGS_. Linear correlation plot between ^166^Ho-only and ^166^Ho-DI with respect to the mean absorbed dose for VOI_HEALTHY_ (**d**), VOI_TUMOR_ (**e**), and VOI_LUNGS_ (**f**), subdivided between pre-treatment (circles) and post-treatment (diamonds), is reported in the bottom row. The solid line depicts linear regression, while the dashed lines indicate the ± 95% confidence intervals

Bland-Altman plots for VOI_HEALTHY_, VOI_TUMOR_, and VOI_LUNGS_ are shown in Fig. 4a–c, respectively. The mean absorbed dose difference between ^166^Ho-DI and ^166^Ho-only was negative for the VOIs within the liver, while it was slightly positive for VOI_LUNGS_. CRP was equal to 5.56 Gy for VOI_HEALTHY_, 17.62 Gy for VOI_TUMOR_, and 2.12 Gy for VOI_LUNGS_.

The linear correlation between ^166^Ho-only and ^166^Ho-DI, assessed using Pearson’s correlation coefficient (depicted in Fig. 4d–f), was equal to 0.99 for VOI_TUMOR_ (for both pre- and post-treatment data)_,_ 0.97 for VOI_HEALTHY_ (*r* = 0.96 for pre- and *r* = 0.98 for post-treatment data), and 0.82 for VOI_LUNGS_ (*r* = 0.72 for pre- and *r* = 0.89 for post-treatment data).

*T* test *p* value results are < 0.00001, 0.00035, and < 0.00001 for VOI_HEALTHY_, VOI_TUMOR_, and VOI_LUNGS_, respectively.

## Discussion

In this study, the qualitative and quantitative accuracy of a ^166^Ho reconstruction derived from a DI acquisition was investigated. The inter-observer agreement (*κ* = 0.093) indicated no specific preference for either the ^166^Ho-only or DI acquisition in the qualitative analysis. The quantitative analysis demonstrated a good correlation between ^166^Ho-only and ^166^Ho-DI with a Pearson’s correlation coefficient > 0.95 for both VOI_HEALTHY_ and VOI_TUMOR_ and 0.82 for VOI_LUNGS_. The difference between mean absorbed dose between ^166^Ho-only and ^166^Ho-DI SPECT was statistically significant for all VOIs (*p* value *<* 0.0005); however, the mean difference was considered clinically not relevant. The limits of agreement for the difference between ^166^Ho-DI and ^166^Ho-only were deemed acceptable by experienced nuclear medicine physicians. Because assessments of dose to the tumor, healthy liver, and lungs serve a different purpose clinically, physicians defined different acceptable limits of agreement for each category prior to this study. A mean difference of 2 Gy with a limit of agreement of ± 5 Gy was considered adequate for healthy liver assessment (median absorbed dose for ^166^Ho-only VOI_HEALTHY_: 27 ± 7.08 Gy), being the dose-limiting factor for radioembolization treatments. A less restrictive value may be applied for the absorbed dose in the tumor because the clinical range for efficacy is variable and not well defined (median absorbed dose for ^166^Ho-only VOI_TUMOR:_: 116 ± 44.91 Gy). With respect to the lungs, according to ^166^Ho-miscrosphere instructions for use [16], a predicted average lung absorbed dose > 30 Gy is a contraindication for the radioembolization treatment. In this study, the clinical acceptable deviation from the difference between ^166^Ho-only and ^166^Ho-DI was defined at approximately 3 Gy. This overestimation prevents underestimation of the lung absorbed dose (median absorbed dose for ^166^Ho-only VOI_LUNGS_: 3 ± 0.99 Gy). For all VOIs, 95% of the data was well within the corresponding clinically acceptable limits of agreement.

Even though rarely encountered (< 1%) [17], radiation pneumonitis is a serious complication that can occur when microspheres inadvertently shunt to the lung parenchyma. So far, lung shunt fraction (LSF) has been the most used metric in clinical routine to determine the activity that shunts to the lungs. Counts in liver and lungs are determined on planar scintigraphy. Despite that the inadequacy of this approach has been demonstrated in multiple studies [18], it is still used in clinical practice. Within the scope of this study, to estimate the difference in the mean absorbed dose between ^166^Ho-DI and on ^166^Ho-only in the VOI_LUNGS_, the lungs were delineated on the corresponding attenuation correction LDCT. However, the lungs were not always entirely visible within the field of view of the SPECT. This drawback is negligible in case the lung perfusion is homogeneous, but this assumption is not always correct [19, 20]. In addition, the very low values of mean absorbed dose in VOI_LUNGS_ were more affected by this drawback. This explains the lower Pearson’s correlation coefficient (0.82) and the higher number of outliers.

The use of the proposed DI protocol has potential benefits, amongst which the possibility to (semi) automatically identify and delineate tumor and healthy tissue within a single SPECT/CT acquisition. Additionally, simultaneous acquisition of both isotopes avoids the registration difficulties, both being time consuming and prone to additional errors in dosimetry.

SPECT images showing either ^99m^Tc distribution or ^166^Ho accumulation can be processed to obtain an automatic delineation of the regions of interest. Healthy liver might be delineated on the ^99m^Tc reconstruction while tumor lesions presenting focused ^166^Ho uptake can be delineated on the ^166^Ho image. The definition of these two compartments is a requirement for the use of the partition model [21]. This method allows the determination of a selective prescribed activity aiming at maximization of the absorbed dose to the tumor tissue, while restricting radiation absorbed dose to the healthy tissue.

Some limitations apply to this study. All images were acquired with the same SPECT/CT scanner, which restricts the used k-factor to this imaging setup. Nonetheless, it is possible to extend this study to other scanners (see Supplemental material). Furthermore, the liver VOIs were manually segmented to ensure an adequate volume definition, similar among all datasets (as can be seen in Table 2), and to avoid the introduction of errors due to thresholding of the ^99m^Tc images. Because of the need to apply the same VOIs to both ^166^Ho-only and ^166^Ho-DI for tumor and healthy liver, a co-registration process was involved. This can lead to small misalignments, which may have impacted on the mean absorbed dose difference. In addition, tumors with a volume smaller than the 25 ml, not considered for this study, could be more affected by registration related error and partial volume effect which will further hamper the tumor dosimetry quantification.

To cope with the mentioned limitations, some future steps can be taken. A phantom experiment could help to determine the accuracy with which it is possible to recover the absorbed dose at different activity concentration, both with and without the presence of ^99m^Tc-colloid. To compensate for the lungs region just partially covered by the SPECT field of view, it could be possible to implement a two-bed position protocol to cover both the lungs and the abdominal region. An assessment of ^99m^Tc crosstalk impact on ^166^Ho acquisitions stratified by volume of interest dimensions could provide a better insight in the possibility to perform ^166^Ho-DI acquisition without being hampered by an increase effect of partial volume effect due to the presence of an additional isotope.

The possibility to skip the ^166^Ho-only SPECT/CT acquisition has a beneficial effect on patients, decreasing the discomfort related to an imaging procedure that takes half an hour. Further analysis is required to implement a clinical workflow to automatically process information derived from the DI protocol and obtain personalized planning for radioembolization.

## Conclusion

Based on a qualitative, as well as a quantitative analysis on patient data, a ^166^Ho-DI SPECT can be safely used instead of a ^166^Ho-only acquisition. The differences between the ^166^Ho-only and ^166^Ho-DI protocol reconstructions were considered to be clinically acceptable, and thus the dual isotope protocol can be adopted in clinical practice.

## Supplementary information

**Additional file 1.** Supplementary materials.

## Data Availability

The datasets used and/or analyzed during the current study are available from the corresponding author on reasonable request.

## Abbreviations

^166^Ho: Holmium-166
^99m^Tc: Technetium-99 m
CRP: Coefficient of reproducibility
DI: Dual-isotope
HEPAR PLuS: Holmium Embolization Particles for Arterial Radiotherapy Plus ^177^Lu-DOTATATE in Salvage NET patients
LDCT: Low dose computed tomography
LoA: Limits of agreement
SPECT: Single photon emission computed tomography
VOI: Volume of interest
