# Supplementary material for: Quantitative 166Ho-microspheres SPECT derived from a dual-isotope acquisition with 99mTc-colloid is clinically feasible
Source: EJNMMI Phys. 2020 Jul 14;7:48. doi: 10.1186/s40658-020-00317-8 (PMC7359973; doi:10.1186/s40658-020-00317-8)
Supplement: Supplementary file 1 — Additional file 1. Supplementary materials. [file 40658_2020_317_MOESM1_ESM.pdf]

Supplementary material for:

Quantitative ^{166}Ho-microspheres SPECT derived from a dual-isotope acquisition with ^{99m}Tc-colloid is clinically feasible

Stella M[1,*], Braat AJAT[1], Lam MGEH[1], de Jong HWAM[1], van Rooij R[1]

[1]Department of Radiology and Nuclear Medicine, University Medical Center, Utrecht, Heidelberglaan 100, 3584 CX Utrecht, The Netherlands

[*]Stella, M.

e-mail: M.Stella@umcutrecht.nl

telephone number: 088-7556295

k-factor analysis

The downscatter in the 81 keV window can be expressed as the sum of the contributions from ^{166}Ho and ^{99m}Tc separately:

$S_{81} = S_{81}^{Tc} + S_{81}^{Ho}$ (I),

where S_{81} is the total downscatter estimate in the 81 keV window and S_{81}^{Tc} and S_{81}^{Ho} represent the individual scatter contributions from ^{99m}Tc and ^{166}Ho respectively. These only include scattered photons originating from higher energy emissions (including bremsstrahlung from the β^- emissions), but not the photopeak scatter (originating from the primary 81 keV photopeak). These contributions can be estimated from the 118 keV window, using their individual k-factors:

$S_{81}^{Tc} = k_{118\to81}^{Tc} * S_{118}^{Tc}$ (II),

and similarly for ^{166}Ho:

$S_{81}^{Ho} = k_{118\to81}^{Ho} * S_{118}^{Ho}$ (III),

where the $k_{E1\to E2}^{A}$ are the k-factors for isotope A to estimate scatter in window E2 from window E1. Because the 118 keV energy window does not cover any gamma emission lines, the projection images recorded in the 118 keV window (P_{118}) are composed of the sum of the scatter contributions S_{118}^{Tc} and S_{118}^{Ho}, and hence, $S_{118}^{Tc} = P_{118} - S_{118}^{Ho}$. Furthermore, since the 170 keV energy window contains counts from ^{166}Ho downscatter exclusively, S_{118}^{Ho} can be estimated from the projection images recorded in the 170 keV window (P_{170}) as: $S_{118}^{Ho} = k_{170\to118}^{Ho} * P_{170}$. Thus S_{118}^{Tc} can be written as $S_{118}^{Tc} = P_{118} - k_{170\to118}^{Ho} * P_{170}$. Substituting in (II) and (III) yields:

$S_{81}^{Tc} = k_{118\to81}^{Tc} * (P_{118} - k_{170\to118}^{Ho} * P_{170})$

and:

$S_{81}^{Ho} = k_{118\to81}^{Ho} * k_{170\to118}^{Ho} * P_{170}$.

After rearranging, equation (I) can be expressed in terms of the projection images recorded in the 118 keV and 170 keV windows:

$S_{81} = k^{Tc}_{118\to81} * P_{118} + (k^{Ho}_{118\to81} - k^{Tc}_{118\to81}) * k^{Ho}_{170\to118} * P_{170}$ (IV)

The factors $k^{Tc}_{118\to81}$ and $k^{Ho}_{170\to118}$ can be estimated from measurement because the windows do not contain any gamma emission lines.

The factor $k^{Ho}_{118\to81}$, however, cannot be measured directly due to the ^{166}Ho photopeak in the 81 keV window. Using a Monte-Carlo simulation, the 81 keV ^{166}Ho gamma emission line can be artificially ignored such that the simulated projections only contain downscatter contributions, similar to the 118 keV window. In previous work, using a digital anthropomorphic phantom (XCAT), $k^{Ho}_{118\to81}$ was simulated to be 1.15 [1]. This method was repeated for this work, but using the patient scans rather than the digital phantom, resulting in a k-factor of 1.17 (see **Tab.4**).

In this work, the factor $k^{Tc}_{118\to81}$ (and $k^{Ho}_{170\to118}$ similarly) was computed by dividing the counts in the 81 keV and 118 keV energy window of ^{99m}Tc (or ^{166}Ho) projections. More specifically, for every projection angle (120 in total) a relevant Region of Interest (ROI) was determined by thresholding the corresponding 140 keV ^{99m}Tc projection image (20% of maximum intensity after smoothing with a 15mm Gaussian filter). The sum of counts within the ROIs in both windows (81 keV and 118 keV) was divided for each angle and a k-factor was determined as the mean over all angles, as shown in **Fig.5 (a)**. This procedure was repeated for 65 patient scans to obtain a median k-factor of $k^{Tc}_{118\to81}$=1.15, as it is marked with the solid line in **Fig.5 (c),** with a quartile deviation equal to 0.05.

Because $k^{Ho}_{118\to81}$ and $k^{Tc}_{118\to81}$ do not differ significantly (see **Tab.4**), Eq. IV can be approximated as:

$S_{81} = k^{Tc}_{118\to81} * P_{118}$.

As the P_{170} contribution is negligible since the $(k^{Ho}_{118\to81} - k^{Tc}_{118\to81}) * k^{Ho}_{170\to118}$ term is equal to 0.01.Therefore, to estimate the downscatter in the 81 keV window, a k-factor of 1.15 can be applied independent of the relative contributions of ^{166}Ho and ^{99m}Tc. However, it is dependent on average patient geometry, definition of energy windows and collimator/detector specifics. The computed k-factors are reported in the **Tab.4** (median values reported for both window width dependent and independent measurements).

References

[1] B. J. van Nierop, J. F. Prince, R. van Rooij, M. A. A. J. van den Bosch, M. G. E. H. Lam, and H. W. A. M. de Jong, "Accuracy of SPECT/CT-based lung dose calculation for Holmium-166 hepatic radioembolization before OSEM convergence," *Med. Phys.*, vol. 45, no. 8, pp. 3871–3879, 2018.

Fig. 5 (**a**) The ratio between counts, due to ^{99m}Tc downscatter, in the 81 keV and 118 keV energy windows, for each acquisition angle, for a single patient. The solid black line represents the mean value over the acquisition angles. (**b**) Box plots indicating the median and interquartile ranges (IQR) of the count ratios for the first 10 patient scans (whiskers indicate the range, excluding points further than 1.5 IQR from the box). (**c**) Mean ratios for all the scans, with a solid line representing the overall median value (1.15), and 1st and 3rd quartiles (dashed lines)

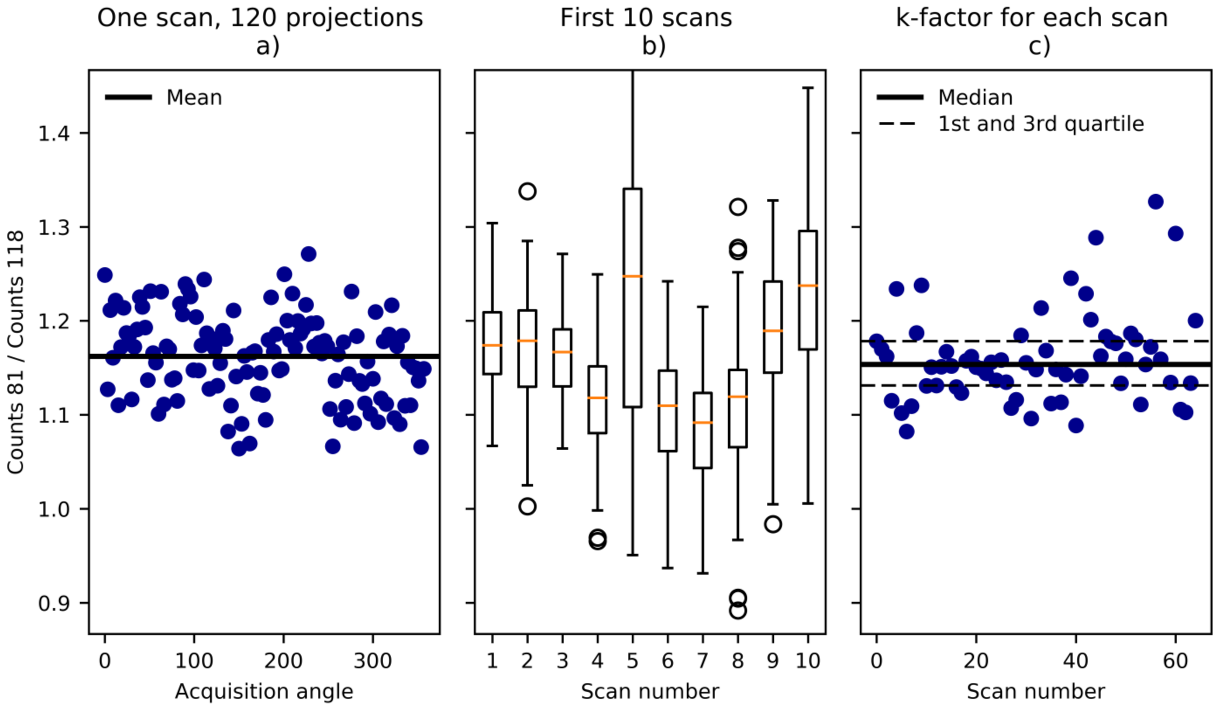

Tab. 4 Median values for the computed k-factors, reported both energy window width dependent (i.e., specific to the window widths applied in this work) and independent. Subscripted values refer to the energy windows considered, the corresponding isotope is superscripted.

	Width dependent	*Width independent*
$k^{Ho}_{170\rightarrow140}$	0.97	0.95
$k^{Ho}_{170\rightarrow118}$	0.61	0.88
$k^{Ho}_{170\rightarrow81}$ *	0.77	1.30
$k^{Ho}_{118\rightarrow81}$ *	1.17	1.37
$k^{Tc}_{118\rightarrow81}$	1.15	1.34

* *Downscatter simulation excluding photopeak*
